# Improving the evidence base for services working with youth at-risk of involvement in the criminal justice system: developing a standardised program approach

**DOI:** 10.1186/s40352-018-0066-5

**Published:** 2018-04-16

**Authors:** Alice Knight, Myfanwy Maple, Anthony Shakeshaft, Bernie Shakehsaft, Tania Pearce

**Affiliations:** 1 National Drug and Alcohol Research Centre (NDARC), Faculty of Medicine, UNSW Australia, Randwick, NSW 2052 Australia; 2 Social Work, School of Health, University of New England, Armidale, NSW Australia; 3 BackTrack, Armidale, NSW Australia

**Keywords:** High-risk young people, Multiple risk behaviour, Young people with multiple and complex needs, Youth program, Complex intervention, Intervention, Evaluation

## Abstract

**Background:**

Young people who engage in multiple risk behaviour (high-risk young people) such as substance abuse, antisocial behaviour, low engagement in education and employment, self-harm or suicide ideation are more likely to experience serious harms later in life including homelessness, incarceration, violence and premature death. In addition to personal disadvantage, these harms represent an avoidable social and economic cost to society. Despite these harms, there is insufficient evidence about how to improve outcomes for high-risk young people. A key reason for this is a lack of standardisation in the way in which programs provided by services are defined and evaluated.

**Methods:**

This paper describes the development of a standardised intervention model for high-risk young people. The model can be used by service providers to achieve greater standardisation across their programs, outcomes and outcome measures. To demonstrate its feasibility, the model is applied to an existing program for high-risk young people.

**Conclusions:**

The development and uptake of a standardised intervention model for these programs will help to more rapidly develop a larger and more rigorous evidence-base to improve outcomes for high-risk young people.

## Background

Adolescence is a period of increased risk for adverse physical and mental health outcomes: approximately half of all lifetime mental disorders, for example, begin by the mid-teens and three quarters by the mid-twenties (Kessler et al. 2007). The majority of young people experience relatively few harms as a consequence of engaging in a small number of risk behaviours, such as recreational substance use and physical inactivity (Australian Institute of Health and Welfare 2011). Nevertheless, a minority of adolescents will experience more substantial harms associated with multiple risk behaviours (high-risk young people), including exposure to violence, homelessness, incarceration, and premature death (Bruun and Mitchell 2012; DHS et al. 2010; Hawkins 2009; Mitchell 2011; United Nations 2004). Moreover, the presence of multiple risk behaviours has a multiplier effect: increased frequency of substance abuse and involvement in crime, for example, is associated with disengagement from school and declining academic performance, which in turn is associated with reduced employment opportunities (as a consequence of criminal convictions and poor literacy) and increased risk of self-harm, suicide, and recidivism into both juvenile and adult prisons (Bruun and Mitchell 2012; Henry 2010; McLaughlin et al. 2010).

Complicating the presence of multiple risk behaviour is that their aetiology is typically complex, being associated with a range of social determinants of poor health, including childhood abuse, low socio-economic status (SES), and minority cultural identity (Fergusson et al. 2000; Groark and McCall 2009; Vitaro and Tremblay 2009). This complex aetiology implies the occurrence of significant harm among young people will not be spread randomly across individuals in a population, but will cluster within defined sub-populations. Indigenous people in Australia, for example, have had a recent history of colonisation (including dispossession from their land), racism, oppression, and low SES which, in turn, has increased the rate with which they experience mental health and physical harms. Alcohol-related suicide rates among 15–29 year old Indigenous Australians, for example, are four (males) and five (females) times higher than for non-Indigenous young people (Calabria et al. 2010), rates of all-cause alcohol-related disease and injury are more than double for Indigenous males and seven times greater for Indigenous females (Calabria et al. 2010), and more than 50% of 10–17 year old juvenile detainees are Indigenous, despite Indigenous Australians comprising an estimated 2–3% of the population (Australian Institute of Criminology 2008; Australian Institute of Health and Wefare 2003).

In addition to personal disadvantage, these harms represent an avoidable social and economic cost to society, including: increased social disruption, such as loss or damage to property and fear for personal safety; increased need for health care at an earlier stage in life (e.g. hospital and rehabilitation services for injury); greater reliance on social security benefits; and avoidable police, court and incarceration costs. The cost of juvenile custodial services in 2010/2011 in one state in Australia (New South Wales) was $114.5 million, for example, while the cost of poor mental health among young people in Australia alone, in terms of employment, health and social impacts, is estimated to cost $6.3 billion per annum (Australian Institute of Criminology 2008; ReachOut Australia, 2015). Youth detention costs are similarly high internationally. The long-term cost of the confinement of young people in the US, including the cost of recidivism, lost educational opportunities, and lost future earnings and taxes, is estimated to be between US$8billion and US$21billion per annum (The Justice Policy Institute 2014). From a lifetime perspective, the monetary value of a 14-year-old high risk juvenile avoiding crime over his/her lifetime was estimated to be between US$3.2 billion and US$5.8 billion (Cohen and Piquero 2009), while the economic burden associated with the entire sub-population of disengaged youth was estimated to be US$4.7 trillion in 2011 (Belfield et al. 2012). In the UK, the average annual cost of detention is estimated to be £65,000 for Youth Offender Institutions, £178,000 for Secure Training Centres, and £212,000 for Secure Children’s Homes (Natale and Williams 2012; The Ministry of Justice 2013), while the UK’s Youth Justice Board estimated the cost of detention for the entire sub-population of detained young offenders was £245 million in 2012/2013 (The Ministry of Justice 2013).

Although the poor personal, social, and economic outcomes experienced by high-risk young people highlights the need for relevant, high-quality intervention programs, a 2016 systematic literature review conducted by the authors found that there are very few published evaluations of programs that simultaneously target multiple risk behaviour in young people: of the 268 relevant studies published in the international literature between 2009 and 2014, only 13 (5%) were evaluations of programs for young people who engaged in multiple risk behaviour, and half of these were rated as methodologically weak against standard criteria (*n* = 7) (Knight et al. 2017). Moreover, of the 13 identified programs, all but one had been implemented in relatively controlled settings (e.g. a school or a health clinic), all used a different model of intervention (e.g. each targeted a different combination of risk behaviours using a different combination of program components), and all quantified the effectiveness of the program using a wide variety of outcome measures (Knight et al. 2017).

The small number of methodologically adequate evaluations of programs means there is limited high-quality evidence that service providers and policy makers can use to improve the effectiveness of programs for high-risk young people. The lack of homogeneity in intervention components, outcomes, and outcome measures limits the ability to use rigorous evaluation designs in determination of program effectiveness, pool results into meta-analysis (as a method of increasing the strength of existing evidence) and reduces the generalisability of the results to other populations of high-risk young people. One way to rapidly engender a larger and more rigorous evidence-base to support the uptake of best evidence programs for high-risk young people, is to achieve greater standardisation in the way in which programs provided by services are defined, implemented, and evaluated.

This paper describes the development of a standardised intervention model that could be used to achieve greater standardisation across programs, outcomes, and outcome measures delivered by different services for high-risk young people. It has two specific aims. First, to describe the development of the model. Second, to apply the model to an existing program for high-risk young people to demonstrate how it can be operationalised and how it might be replicated by other programs.

## Methods

### The development of a standardised intervention model

As delineated in Table 1, the proposed standardised intervention model adapts a program logic framework to ensure clarity about the proposed program components (part b), why they are likely to effective (part c – mechanisms of change), and to ensure the program components are strongly aligned with the specific problems being targeted (part a), the outcomes and outcome measures (part d), and the process measures (part e) (Dalkin et al. 2015; Groark and McCall 2009). While the program logic concept per se is not new, this proposed standardised intervention model has two key innovations. First, it includes stipulating a mechanism of change (part c) which requires a clear articulation of the rationale for change: that is, why the proposed program (the core components and flexible activities) would be expected to achieve the proposed outcomes. The primary purpose of the mechanism of change is to challenge those designing new, or refining existing, programs to be clear about exactly what outcomes each program component is attempting to achieve. Second, the development of the proposed program components (part b) allows programs for high-risk young people to be both standardised (the core components) and adaptable to the individual circumstances of different services (the flexible activities), as opposed to the more narrow and rigid way in which programs have been typically defined, which limits their generalisability and comparability (Knight et al. 2017). This standardised but flexible approach is aimed at solving the well-established, but as yet difficult to resolve, tension articulated in the complex intervention literature, between the need for standardisation (to provide adequate comparability across programs delivered by different services in different circumstances) and the need for sufficient flexibility (to allow tailoring to the resources and circumstances of different settings (N. C. Campbell et al. 2007; Craig et al. 2008; Hawe et al. 2004). The bolded text in Table 1 are the components of the proposed standardised intervention model (aim 1), while the normal text highlights how it is tailored to the specific circumstances of one program (aim 2).

**Table 1:** The proposed standardised intervention model components (bold text) and its application to the BackTrack program (normal text)

a. Areas of need	b. Intervention		c. Mechanisms of change	d. Outcomes (outcome measures)	e. Process measures
	Core components	Flexible activities			
**- Emerging or established involvement in criminal incidents and the criminal justice system** **- Tenuous engagement with the education system and/or un-, under-employment** **- Risky drug and alcohol use** **- Low self-efficacy and/or emerging mental health issues**	**1. Engagement**	**-** Paws-Up **-** Youth forum	**1. Successful engagement with participants ensures sufficient exposure to program components**	**-** ***A reduction in crime/severity of crime*** **(e.g. routinely collected police incident data; self-reported involvement in crime)** **-** ***A reduction in substance misuse*** **(e.g. Alcohol Use Disorder Identification Test [AUDIT], the Alcohol, Smoking and Substance Involvement Screening Test [ASSIST], the Heaviness of Smoking Index [HSI])** **-** ***A reduction in suicide ideation and/or psychological distress*** **(e.g. self-reported suicide ideation; psychological distress [such as Kessler-6])** **-** ***Improved employment options*** **(e.g. employment status; school attendance; formal skills training; work experience)** **-** ***Improved self-efficacy or resilience*** **(e.g. the Connor-Davidson Resilience Scale)**	**- The extent to which the program was delivered as planned (program fidelity)** **- Participant attendance and exposure to the different core components of the program (program dose)** **- Participant satisfaction with the program** **- Participant acceptability of the program** **- Contextual facilitators/barriers to program implementation**
	**2. Case management**	**-** Assist with legal issues (e.g attend court) **-** Work ready preparation **-** Contingency planning **-** Inter-agency liaison	**2. Prioritising participants’ most immediate problems (e.g. legal issues), and developing pragmatic solutions to these problems, allows participants to focus on pro-social activities**		
	**3. Diversionary activities**	**-** Supervised events in town on weekends **-** Interstate travel on weekends to community events (e.g. Dog jump competitions) **-** Day-to-day attendance at the program	**3. Reducing participants’ exposure to high-risk situations (at home and in public), at high-risk times (e.g. the weekend)**		
	**4. Personal development, identity, and team identity**	**-** Circle Work **-** Chilling the brain **-** Counselling **-** BackTrack shirts	**4. Improving participants’ capacity to manage when they are in high-risk situations**		
	**5. Training and skill development**	**-** BackTrack school **-** Work experience **-** Vocational training **-** Volunteer work experience	**5. Improving participants’ education and life skills to increase their opportunities for active participation in employment**		

The development of this standardised intervention model required establishing the core, standardised program components that would need to be delivered by any program for high-risk young people (whilst the flexible activities that operationalise these core components are, by definition, the responsibility of each service to articulate). Five standardised, core program components were developed using the central tenet of evidence-based practice: that is, by integrating the best available external evidence with the expertise of individual service providers (Sackett et al. 1996). The best available external evidence was distilled by the 2016 systematic review conducted by the authors (Knight et al. 2017), and the expertise of service providers was obtained through the process of applying the initial model (i.e. the first draft of the model that was based only on the published literature) to an existing program.

### The best available external evidence

The systematic review identified four commonalities across published evaluations of programs. First, the 13 evaluated programs targeted a mean of three risk behaviours, ranging from two to six per program: no program targeted a single risk behaviour (Knight et al. 2017). This highlights the need for programs to comprise multiple components aimed at addressing participants’ multiple risk behaviour. Second, a detailed critique of the six evaluations identified as being of moderate or good methodological quality identified three common core components: i) *case management*, to help young people navigate the pressures of their day-to-day lives; ii) utilising behaviour change techniques to foster *personal development* and assist the young people to better understand their thoughts and behaviours; and iii) providing access to *training and/or skill development* to increase their chances of accessing meaningful employment.

More specifically, *case management* requires a high degree of cooperation and communication between different service providers in the community, and highlights the importance of, as far as possible, having the same case worker or case manager. The prioritisation of the most immediate problems being experienced by a young person, and identifying pragmatic solutions for these problems, such as securing crisis accommodation or facilitating access to legal aid for court appearances, were identified as a critical focus for case management. *Personal Development* was fostered through the application of evidence-based behaviour change techniques: of the six programs evaluated, three primarily used Motivational Interviewing (MI) techniques (Bannink et al. 2014; Cunningham et al. 2012; Mason et al. 2011), two primarily used Cognitive Behavioural Therapy (CBT) (Poirier et al. 2013; Rohde et al. 2012), and one primarily used Multisystemic Therapy (MST), Multidimensional Family Therapy (MDFT) or Functional Family Therapy (FFT) (Schaeffer et al. 2014). *Training and/or skill development* was used to different extents. The three intervention programs that implemented MI techniques, for example, provided tailored information to participants on specific risk behaviours, in an effort to improve their understanding of the risk behaviour and their skills to modify their behaviour. Two programs explicitly provided opportunities for active participation in education or training (e.g. classroom-based skill development, numeracy and literacy training, employability training, study skills and schoolwork techniques, or work experience) to improve participants’ chances of securing employment.

### The expertise of individual service providers

The research team facilitated two workshops with staff from an existing program for high-risk young people to obtain their input into the development of the standardised intervention model. These workshops were held at the University of New England (UNE) in March and May 2014. The primary purpose of the first workshop was to report the key findings from the critical review of the literature (the best available external evidence) and examine their relevance to their program. The primary purpose of the second workshop was to map their current service delivery model to the first version of the standardised intervention model that was based solely on the findings from the critical review. These workshops identified two additional program components that staff perceived as being critical to their approach to working effectively with high-risk young people. The first, *engagement*, recognises that success in the program is largely determined by the extent to which participants are actively engaged with the program, and to increase the likelihood that they attend for enough time to gain sufficient exposure to the program components. To enhance engagement, staff emphasised the importance of voluntary participation, and ensuring that young people have the opportunity to choose to participate and take ownership of their decisions. The second additional component, *Diversionary Activities*, was included after staff highlighted the importance of needing to divert high-risk young people from high-risk activities and peers (e.g. antisocial behaviour in public places) during high-risk times (e.g. late at night or during the weekends), in order to achieve both reduced short-term exposure to high-risk situations and sustained behaviour change.

Further to identifying these additional two core program components, the workshops with the service providers was used to articulate the mechanism of change for each core component: i) *effective engagement* ensures participants are exposed to a sufficient number of intervention components; ii) *case management* ensures participants’ most immediate problems are prioritised (e.g. legal issues); iii) *diversionary activities* reduce participants’ exposure to high-risk situations at high-risk times (e.g. late at night or on the weekend); iv) *personal development, identity, and team identity* improve participants’ capacity to manage when they are in high-risk situations and create a sense of belonging and acceptance; and v) *learning and skills development* increase the opportunities for active participation in employment and greater engagement with their communities.

### The application of a standardised intervention model to an existing program for high-risk young people

To demonstrate the feasibility of operationalising the proposed standardised intervention model delineated in Table 1, it was applied to an existing program for high-risk young people called BackTrack.

### Overview of the BackTrack program

The BackTrack program was established in Armidale in northern New South Wales (NSW) in 2006 (http://www.backtrack.org.au). It is underpinned by six key principles: i) the need for multiple program components, which recognises that participants are more likely to engage in multiple risk behaviours which can be targeted simultaneously (e.g. personal development, skills training and legal issues); ii) flexibility in the delivery of the program components, which reflects that the focus of young people’s needs shifts over time; iii) flexibility in program attendance, so that participants are able to start, leave and re-enter the program as they wish, or their life circumstances permit; iv) a requirement that young people in the program eventually actively participate in all components of the program; v) active engagement of local businesses, local media, key stakeholders (e.g. police, magistrates), and community members in delivering program elements, resolving bureaucratic problems, providing infrastructure and funds, and facilitating communication about the benefits of the program; and vi) recognition that achieving sustained change among high-risk young people will take a number of years.

### Applying the standardised core components to BackTrack

The three common themes identified in the existing literature, and the additional two components identified by staff in the workshops, became the foundation for the multiple core program components within the standardised intervention model, and were used to guide the classification of existing BackTrack program activities. For example, where staff described learning activities they implemented with participants to improve their literacy and numeracy skills, these were classified as belonging to the core component of *Training and Skill Development*. A brief description of the BackTrack program activities, as they relate to the five program components, is provided below.

### Core component 1: Engagement

The major engagement activity for BackTrack is called ‘PawsUp’. It involves participants initially interacting with working dogs, in terms of simple unstructured play and involvement in their care. A second engagement activity is called the ‘Youth Forum.’ This is led by existing participants, rather than staff, and requires all new participants to agree to the ground rules of BackTrack. It specifies the consequences of failing to meet these ground rules. All participants are encouraged to recognise the difficulties that they each face in their lives and to support each other to make BackTrack work for them, despite coming from a range of different schools, neighbourhoods, communities and cultural backgrounds.

### Core component 2: Case management

The highest priority issues in the first year of BackTrack participation are typically related to legal and mental health issues. Consequently, staff will work with participants to ensure that participants have access to resources and meet their obligations (e.g. accessing Legal Aid, attending court on time in clean clothes, advocating to the magistrate on their behalf, and providing formal reports for court). This focus is combined with group tutorials on how the legal system works, and informal discussions at BackTrack, periodically attended by local police and the local Magistrate. Over time, the specific range of case management activities typically shifts from a focus on acute legal issues to improved educational attainment and employability. These activities include: ‘work ready preparation’ (e.g. obtaining a Tax File Number, opening and managing bank accounts, arranging appropriate transportation to work); contingency planning (supporting participants to manage challenging situations that occur in their day-to-day lives, such as housing insecurity and health issues); and, inter-agency liaison (developing and maintaining relationships with a range of agencies and key stakeholders to minimise risky situations, and optimise opportunities for training and skill development, personal development, and community integration).

### Core component 3: Diversionary activities

These activities can range from supervised events in town on the weekend or in the evenings, such as trips to the town pool or local football games, to group trips away from town on the weekends, such as camping or to participate in dog-jumping competitions. Day-to-day attendance at the program is also considered an important diversionary activity as participants are engaged in meaningful activity and surrounded by supportive peers and staff. This reduces the likelihood of them becoming bored and helps reduce their interaction with high-risk peer or family members.

### Core component 4: Personal development, identity, and team identity

Many activities within this component draw on elements of motivational interviewing (Naar-King 2011), cognitive-behavioural therapy (Spirito et al. 2011), choice theory (participants can choose activities, for example, and not be concerned about being excluded from the program) (Walter et al. 2008), and mindfulness (Schonert-Reichl and Lawlor 2010). One specific activity BackTrack implements is called ‘Circle Work’ which provides participants the opportunity to verbalise their feelings, instigate conversations about issues with which they are having difficulty coping, and express their hopes for the future. Other activities in this component include anger management, role-playing, mindfulness activities and regular meditation (referred to as ‘chilling the brain’). These activities are applicable to both individuals and the group and can be integrated into BackTrack’s day-to-day activities (e.g. chilling the brain might occur in a mini-bus on the way home from a skills-based activity).

In addition to personal development, activities within this core component provide opportunities for participants to develop a greater sense of belonging to the BackTrack team. One simple activity that operationalises this component is the provision of a distinctive BackTrack shirt, which participants are required to keep clean and wear when they are involved in skills training and community-based activities. For some participants, BackTrack is the only aspect of their lives in which they can develop a sense of pride, achievement and responsibility for their own behaviour, which can become associated with their BackTrack shirt. Since Aboriginal Australians are over-represented in BackTrack (they represent 49% of participants despite comprising only 9% of the local population (Australian Bureau of Statistics 2011)), cultural awareness is also embedded into all program components. Agricultural work, for example, provides an opportunity for discussion with local Aboriginal Elders about Aboriginal and non-Aboriginal methods of land management, and how these might become more closely aligned. The non-Aboriginal participants are routinely engaged in the cultural awareness activities, which builds their understanding of the long history of Aboriginal stewardship and the unique status of Aboriginal Australians as the oldest continuing culture on Earth.

### Core component 5: Training and skill development

BackTrack has partnered with different agencies to provide a range of skill learning options. One example is the BackTrack School, which is taught by a qualified teacher, with a focus on developing basic literacy and numeracy skills. Although the content of the lessons is fixed, because they are legally required to be mapped to the NSW school curriculum, the format of their delivery is flexible to account for participants’ concentration capacity: participants determine the length of lessons, the nature of activities which intersperse lessons (e.g. outdoor exercise or music), and the learning aids that they prefer to use (e.g. participants are encouraged to help each other with tasks and to use the ‘PawsUp’ dogs as reading partners so they are less threatened by their perceived poor literacy).

To complement the BackTrack School, pragmatic skills-based programs are provided in partnership with formal vocational training organisations so that young people build demonstrable, industry recognised, qualifications to improve their employability. Although the specific range of programs provided varies depending on the availability of resources and different vocational training partners, the core set of programs focus on agricultural-related skills because BackTrack is located in a rural community and the programs are designed to meet known skill shortages in the region (to optimise the likelihood that program participants will progress into employment). One skills program, called ‘AgLads’, requires participants to enrol in the agricultural Certificate I and II courses at the local technical college. Another program, called ‘IronMan Welding’, uses an on-site, fully operational welding workshop to develop skills in artistic and functional welding. Art pieces are sold in local markets and at the BackTrack shed location, while the functional components provide metal fabrication products and services for local industry, businesses, and individuals. This program requires participant enrolment in the Certificate II in Metals and Engineering course at the local technical college. Other programs have the same structure (ie: skills based and require enrolment in the relevant course at the local technical college) and focus on developing a range of other recognised skills, including first aid, occupational health and safety, small motor operation and maintenance (e.g. chainsaws and lawn mowers), and operating heavy machinery. In urban settings, programs could develop skills to meet workforce shortages in other sectors of the economy, such as hospitality, manufacturing, and retail.

To avoid the development of skills in isolation from local farmers, industries, businesses, government, and non-government organisations, and to increase the number and strength of connections between participants and their community, BackTrack also actively seeks to create a range of potential job and work experience opportunities for participants. For example, significant flooding in 2012 provided opportunities for BackTrack participants to apply their rural skills on a volunteer basis to assist farmers to repair damage to their properties and minimise their stock losses, while bushfires in 2013 and 2015 provided an opportunity for BackTrack participants to act as refuelling volunteers for fire-fighting helicopters at the local airport (Harris 2015; Meldrum-Hanna 2011; Yamamoto et al. 2013). Volunteering for these activities emphasises the importance of contributing to their community, and provides an opportunity to develop participants’ interpersonal skills, such as teaching them to look directly at people when being introduced and to shake hands as appropriate ways of interacting with others. They also allow participants to gain these skills as a group, so they can support each other in these unfamiliar situations that they find extremely challenging. Utilising these opportunities is a clear example of the process of tailoring program activities to local circumstances, while maintaining the core program component of learning and skills development.

## Discussion

This paper proposes a standardised, best-evidence intervention model that can be used by different services that provide programs for high-risk young people. Given the small number of high-quality evaluations of programs for high-risk young people that have been published in the peer-review literature, and the extent of heterogeneity of both the type of programs available and the outcome measures used to evaluate their effectiveness, increasing the extent of standardisation across programs internationally would build the evidence base by improving the ability to compare seemingly different programs across communities. This option is especially important for these programs because individually, they typically engage with a relatively small number of high-risk young people. BackTrack, for example, only engaged 61 participants between December 2012 and June 2015. The reality of engaging a small number of participants is that it limits the ability to use rigorous evaluation designs, such as a randomised controlled trial (RCT) or the multiple baseline design (MBD) (Hawkins et al. 2007) in any determination of program effectiveness, and it reduces the statistical power of outcome analyses that could be achieved in the evaluation of any one program. A further benefit to standardisation is that it would increase the frequency with which participants’ outcomes are assessed using best-evidence measures and facilitate the pooling of results across studies in meta-analysis.

This paper proposes a pragmatic solution to key methodological limitations that are common across programs for high-risk young people. It describes an intervention model that can be standardised across services, primarily by using five common core program components that are operationalised by service-specific activities. Built on the principles of complex interventions (Bonell et al. 2012; M. Campbell et al. 2000; Craig et al. 2008; Hawe et al. 2004), this model does not require that programs adhere to a prescribed set of intervention activities, but provides a common framework, within which different services can develop and implement their preferred program activities. Although this approach does require the adoption of the five core components to achieve adequate standardisation across programs (as summarised in Table 1), individual programs would still be required to determine their own program activities to operationalise the core components, and could even add their own core components. A cultural connectedness or awareness component, for example, might be highly valued by programs delivered in Indigenous-specific settings, or specifically for minority cultural groups.

Adoption of the intervention model delineated in Table 1 could also help standardise the outcome measures used to assess the impact of different programs. Ideally, these measures would be embedded into the intake assessment procedures of service providers so that high-quality data are collected routinely for all program participants. Programs could augment this standard set of assessment measures with additional measures of relevance to their program. The intake assessment would need to be repeated at agreed time intervals (e.g. three, six and twelve months, then annually thereafter), and although this may impose a task on staff in addition to their regulatory reporting requirements, it could be used to provide personalised feedback to participants on their progress over time, as well as generating comparable measures of the effectiveness of programs.

At the same time that programs are routinely collecting these self-report data, researchers could develop measures of the community-level benefits of programs (e.g. reduced population rates of crime, which might occur if the high-risk young people in a community are associated with the majority of crime committed by young people in a community), as well as methods for routinely conducting economic evaluations to weigh the benefits of programs against their costs. The need for economic evaluations of these programs seems especially acute, given current systematic reviews by the authors did not identify any economic evaluations of programs for high-risk young people (Knight et al. 2017).

## Conclusion

There is a clear lack of rigorous evidence to support the international uptake of programs for high-risk young people (Knight et al. 2017). This paper provides a mechanism for improving this evidence base by increasing standardisation across program components and outcome measures. It proposes a standardised intervention model comprising five core components that are required to be operationalised by individual services, by tailoring them to their available resources and practical circumstances. The feasibility of this process is demonstrated by its application to the BackTrack program. Nevertheless, given staff are likely to have a strong preference for their own, existing program, a key issue is the extent to which program providers are willing to adapt their programs to use the same core program components and the same core assessment tools, in order to achieve a substantially improved evidence-base for these programs in a relatively short period of time through pooled analysis of outcomes from their individual programs. The alternative to adopting this standardised but flexible model is likely to be a continuation of the publication of a small number of under-powered evaluations of varying methodological quality (Knight et al. 2017).

A key next step in improving the evidence-base for TSO-delivered programs for high-risk young people would be to quantify the benefits of at least one program defined using this framework delivered in at least one community (Semczuk, 2015). Given those findings were promising, then the benefits and costs of delivering this model in multiple communities could be estimated, which would strengthen the causal link between the intervention framework and the observed outcomes. Next, this framework could be evaluated when it is delivered by multiple TSOs in multiple communities, which would further strengthen the quality of the evidence-base and the generalisability of the model because it would be informed by the expertise of multiple TSOs. Finally, this larger and more rigorous evidence-base could be used to accelerate the wider uptake of these programs which would, consequently, improve the social, health, and economic outcomes of a greater number of high-risk young people.
